# The endometrial microbiota of women with or without a live birth within 12 months after a first failed IVF/ICSI cycle

**DOI:** 10.1038/s41598-023-30591-2

**Published:** 2023-03-01

**Authors:** Bich Ngoc Bui, Nienke van Hoogenhuijze, Marco Viveen, Femke Mol, Gijs Teklenburg, Jan-Peter de Bruin, Dagmar Besselink, Linda Stevens Brentjens, Shari Mackens, Malbert R. C. Rogers, Gaby S. Steba, Frank Broekmans, Fernanda L. Paganelli, Janneke H. H. M. van de Wijgert

**Affiliations:** 1 Department of Gynaecology and Reproductive Medicine, University Medical Centre Utrecht, Heidelberglaan 100, 3584 CX Utrecht, The Netherlands; 2 Department of Medical Microbiology, University Medical Centre Utrecht, Heidelberglaan 100, 3584 CX Utrecht, The Netherlands; 3 Center for Reproductive Medicine, Reproduction and Development, Amsterdam University Medical Centre, University of Amsterdam, Meibergdreef 9, 1105 AZ Amsterdam, The Netherlands; 4 Isala Fertility Clinic, Isala Hospital, Dokter Van Heesweg 2, 8025 AB Zwolle, The Netherlands; 5 Department of Obstetrics and Gynaecology, Jeroen Bosch Hospital, Henri Dunantstraat 1, 5223 GZ ’s-Hertogenbosch, The Netherlands; 6 Department of Obstetrics and Gynaecology, Radboud University Medical Centre, Geert Grooteplein Zuid 10, 6525 GA Nijmegen, The Netherlands; 7 Department of Obstetrics and Gynaecology, Maastricht University Medical Centre, P. Debyelaan 25, 6229 HX Maastricht, The Netherlands; 8 Brussels IVF, Universitair Ziekenhuis Brussel, Vrije Universiteit Brussel, Laarbeeklaan 101, 1090 Brussels, Belgium; 9 Julius Center for Health Sciences and Primary Care, Utrecht University, Universiteitsweg 100, 3584 CX Utrecht, The Netherlands; 10 UMC Utrecht, Huispostnummer F.05.126, Postbus 85500, 3508 GA Utrecht, The Netherlands

**Keywords:** Microbiology, Infertility

## Abstract

The endometrial microbiota composition may be associated with implantation success. However, a ‘core’ composition has not yet been defined. This exploratory study analysed the endometrial microbiota by 16S rRNA sequencing (V1–V2 region) of 141 infertile women whose first IVF/ICSI cycle failed and compared the microbiota profiles of women with and without a live birth within 12 months of follow-up, and by infertility cause and type. *Lactobacillus* was the most abundant genus in the majority of samples. Women with a live birth compared to those without had significantly higher *Lactobacillus crispatus* relative abundance (RA) (p = 0.029), and a smaller proportion of them had ≤ 10% *L. crispatus* RA (42.1% and 70.4%, respectively; p = 0.015). A smaller proportion of women in the male factor infertility group had ≤ 10% *L. crispatus* RA compared to women in the unexplained and other infertility causes groups combined (p = 0.030). Women with primary infertility compared to secondary infertility had significantly higher *L. crispatus* RA (p = 0.004); lower proportions of them had ≤ 10% *L. crispatus* RA (p = 0.009) and > 10% *Gardnerella vaginalis* RA (p = 0.019). In conclusion, IVF/ICSI success may be associated with *L. crispatus* RA and secondary infertility with endometrial dysbiosis, more often than primary infertility. These hypotheses should be tested in rigorous well-powered longitudinal studies.

## Introduction

The uterus has long been considered a sterile environment. However, evidence for the presence of bacteria, albeit in low quantities, is mounting: next-generation sequencing (NGS) studies have detected bacteria in the uterine cavity belonging to a wide range of phyla, among which *Actinobacteria*, *Firmicutes*, *Bacteroidetes*, and *Proteobacteria*^1–8^. Embryo implantation occurs in the endometrium, and it has been hypothesized that the composition of the endometrial microbiota may affect implantation via modulation of local immune responses and tissues^9,10^. The endometrium is difficult to access and most studies to date have therefore used the vaginal microbiota as a proxy of the endometrial microbiota. In women undergoing assisted reproductive technologies (ART), a *Lactobacillus*-dominated vaginal microbiota profile has been associated with a higher pregnancy probability and the presence of vaginal bacterial vaginosis (BV)-associated anaerobic bacteria with a higher ART failure probability^11–14^. More recently, associations between endometrial microbiota that were not *Lactobacillus*-dominated and poor reproductive outcomes in patients undergoing ART have also been demonstrated^8^. While vaginal microbiota profiling has been proposed as a means to predict ART outcome^15–17^, further research is required to investigate its clinical utility. In addition, new evidence-based methods to improve reproductive outcomes in infertile women and couples are urgently needed because implantation failure accounts for more than 70% of all ART failures^18^.

In this exploratory study, we used 16S rRNA sequencing to characterise bacteria present in endometrial tissue samples of women who had one full failed in vitro fertilization (IVF)/intracytoplasmic sperm injection (ICSI) cycle and were about to undergo a second cycle. Our primary objective was to compare the microbiota profiles of women with and without a live birth within 12 months of follow-up. Our secondary objectives were to compare endometrial microbiota profiles of women with different causes and types of infertility.

## Methods

### Study population and design

**Figure 1:**
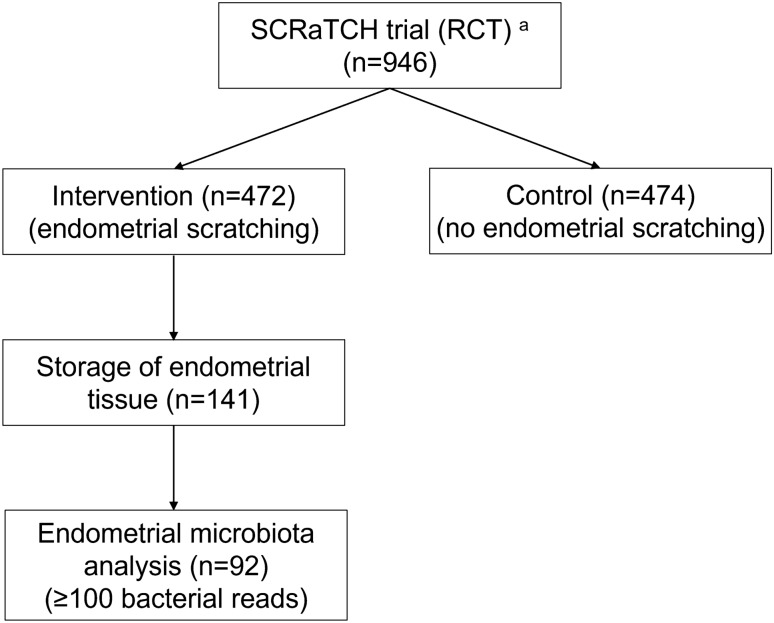
Flow chart of sample selection for the current endometrial microbiota study. RCT, randomised controlled trial. ^a^Endometrial biopsies were obtained from women undergoing endometrial scratching in the SCRaTCH trial, a randomised controlled trial on endometrial scratching in women with a first failed IVF/ICSI cycle^19^.

In the SCRaTCH trial, 472 infertile women were randomised to endometrial scratching and 474 to no intervention. No statistically significant differences in pregnancy outcomes were observed between the groups^19^. The full eligibility criteria of the trial have been described elsewhere^19^. Briefly, women were eligible if they were aged 18–44 years, had failed implantation after one full IVF/ICSI cycle, and were planning to undergo a second full IVF/ICSI cycle. Only 141 of the 472 women in the endometrial scratching group provided informed consent for endometrial tissue storage and future use in research (Fig. 1). We used the tissue samples from all 141 consenting women, which were obtained in the natural cycle prior to their second IVF/ICSI cycle. All women were followed-up for 12 months after randomisation. Women who reached an ongoing pregnancy (defined as a positive heartbeat on ultrasound at 10 weeks gestational age) within those 12 months were followed-up until delivery. The primary outcome was live birth, which was defined as the delivery of at least one live foetus after at least 24 weeks of gestation^19^.

### Tissue sampling and storage

The endometrial biopsy was performed in the mid-luteal phase of the natural cycle prior to the second IVF/ICSI cycle, five to eight days after the luteinizing hormone surge was detected by urinary tests. Endometrial tissue was obtained with an endometrial biopsy catheter (e.g. a Pipelle or other similar catheter). Women were instructed to prevent pregnancy during that cycle by refraining from sexual intercourse or using condoms. They were asked whether they had adhered to this instruction prior to undergoing the endometrial biopsy, and if there was a chance that they could be pregnant, the biopsy was not performed.

Biopsies were performed in an outpatient setting in six hospitals in The Netherlands. All hospitals followed the same protocol for endometrial biopsy and subsequent tissue storage as described below. Physicians wore a sterile gown, sterile gloves and a hair cap to limit microbial contamination as much as possible. All instruments and materials were unpacked from their sterile casing right before the procedure and were put on a sterile field. After insertion of a Trelat speculum, the cervix was extensively washed with sterile water. The Pipelle catheter was then introduced through the cervix up to the uterine fundus, during which care was taken to avoid contact with the vaginal walls. The piston of the catheter was drawn back to the end of the catheter to create a vacuum, and the catheter was slowly retracted within 1–2 min while constantly rotating 360°. After the procedure, physicians put on new sterile gloves and divided the tissue over three tissue tubes (Brooks Life Sciences, USA) using a sterile scalpel and forceps. Within three minutes after taking the biopsy, the tubes were snap-frozen in liquid nitrogen and stored in a − 80 °C freezer as soon as possible.

### DNA isolation, 16S rRNA sequencing and bioinformatics

Frozen endometrial tissue was thawed and homogenized, and total DNA was extracted by bead-beating and chemical lysis (“Supplementary Methods”). The V1-V2 region of the 16S rRNA gene was amplified and sequenced on a MiSeq platform (Illumina, USA). The QIIME2 microbial community analysis pipeline (version 2018.8)^20^ was used with DADA2^21^ for amplicon sequence variant detection, and SILVA as the 16S rRNA reference gene database (SILVA 138)^22^. Reads classified as human DNA, mitochondria or ‘unassigned’ were removed, and data from all samples with < 100 bacterial reads were discarded. The pipeline included positive (mock community) and negative (blank) controls (“Supplementary Methods”).

### Data analysis

In our primary analysis, we compared women with a live birth (from ongoing pregnancies occurring within 12 months after randomisation in the SCRaTCH trial) to women with no live birth. Within the no live birth group, we also compared women with no pregnancy to women with pregnancy loss. No pregnancy was defined as never having had beta-hCG detected in serum or urine. Pregnancy loss was defined as loss of an intrauterine pregnancy or loss of a pregnancy after beta-hCG had been detected in serum or urine but before ultrasound evaluation ≤ 24 weeks of gestation, excluding ectopic pregnancies. Our secondary analyses included comparisons of different infertility causes (categorised as male factor infertility, unexplained infertility, and other infertility causes) and infertility types (primary versus secondary infertility). Male factor infertility was defined as a semen analysis with total motile sperm count of < 10 million^23^. Unexplained infertility was defined as the inability to conceive after at least one year of unprotected intercourse while having ovulatory menstrual cycles (defined as a mean cycle length of 21–35 days), at least one patent fallopian tube (i.e. a negative *Chlamydia trachomatis* antibody titre and/or evidenced by hysterosalpingography or laparoscopy), and a partner with normal semen analysis by WHO criteria^24^. The group of other infertility causes included mostly women with a female cause, such as tubal factor, ovulatory disorder or endometriosis, but also included women with mixed causes (e.g. male and female causes). We hypothesized that the endometrial microbiota composition as a potential infertility cause was least likely in the male factor and other causes of infertility groups because the infertility cause was known in these cases, and most likely in the unexplained infertility group. Additionally, the group of other causes of infertility was small and heterogeneous. We therefore compared: (1) the male factor infertility group with the unexplained infertility group (excluding the other causes group); (2) the male factor infertility group with the unexplained infertility plus other causes groups combined; and (3) the unexplained infertility group with the male factor infertility plus other causes groups combined.

In addition to untargeted analysis of the entire taxonomic table, we also categorised the microbiota data into biologically meaningful ways for use in targeted analyses. First, we grouped the relative abundances (RAs) of related taxa together into the following bacterial groups: (1) *Lactobacillus* genus (consisting of the subgroups *L. crispatus*, *L. iners*, and other lactobacilli), (2) *Gardnerella vaginalis*, (3) other bacterial vaginosis (BV)-associated anaerobes, and (4) a residual group of ‘other bacteria’. Supplement 2 contains an overview of all taxa that were identified in the study and how we grouped them. Second, we categorised samples as containing an RA of ≤ 10%, 11–89%, or ≥ 90% total *Lactobacillus* or a *Lactobacillus* subgroup. The non-lactobacilli bacterial groups were categorized as ≤ 10% or > 10%.

Continuous data were compared using Wilcoxon rank sum tests because they were considered non-normally distributed. Categorical data were compared using Fisher’s exact tests. Alpha diversity was described by inverse Simpson and Chao1 indices and beta diversity by principal component analysis (PCA) based on centered log-ratio (clr) data transformation. ANCOM-BC with false discovery rate (FDR) adjustment for multiple testing was used for untargeted detection of differentially relatively abundant taxa^25^. Differences in RAs of prespecified bacterial groups in targeted analyses were determined by Wilcoxon rank sum tests. Multivariable modelling was not performed due to the small sample size. We used R 4.1.0 (phyloseq^26^ and microbiome^27^ packages) and IBM SPSS Statistics version 26.0 for all analyses.

### Ethics approval

The SCRaTCH trial, a randomised controlled trial from which the endometrial tissue samples were obtained, was approved by the Institutional Review Board of the University Medical Center Utrecht (approval number 15-495, 30 November 2015) (12). For the current microbiota study, ethical approval was obtained from the Biobank Research Ethics Committee of the University Medical Centre Utrecht (approval number 19-520, 31 October 2019). All methods were performed in accordance with the relevant guidelines and regulations.

## Results

### Participant characteristics

The median age of the 141 women was 35.7 years and their median duration of infertility was 30 months (Table 1). During the 12-months follow-up period, 84/141 women (57.4%) conceived and 61/84 of them (72.6%) reached a live birth. Of the women who reached a live birth, 1/61 (1.6%) conceived spontanously and 60/61 (98.4%) with IVF/ICSI.

**Table 1:** Characteristics of all participants and by number of bacterial reads (≥ 100 and < 100).

	All participants (n = 141)	≥ 100 reads (n = 92)	< 100 reads^a^ (n = 49)	P ≥ 100 vs. < 100 reads^b^
Median female age in years (IQR)	35.7 (31.9–39.4)	34.9 (31.2–39.4)	36.5 (33.9–39.8)	0.055
Median female BMI in kg/m^2^ (IQR) ^c^	23.8 (21.6–26.4)	23.5 (21.8–25.6)	24.1 (21.4–27.9)	0.214
Median duration of infertility in months (IQR)	30.0 (22.5–43.0)	29.5 (23.0–43.0)	31.0 (20.0–43.5)	0.952
Female smokers, n (%) ^d^	18 (12.8)	8 (8.7)	10 (20.4)	0.061
Type of infertility of female, n (%) ^e^				0.157
Primary	73 (51.8)	52 (56.5)	21 (42.9)	
Secondary	68 (48.2)	40 (43.5)	28 (57.1)	
Cause of infertility, n (%)				0.929
Male factor	69 (48.9)	46 (50.0)	23 (46.9)	
Unexplained	60 (42.6)	38 (41.3)	22 (44.9)	
Other^f^	12 (8.5)	8 (12.7)	4 (8.2)	
Median # previous embryo transfers per participant (IQR)	2.0 (1.0–3.0)	2.0 (1.0–3.0)	2.0 (1.0–3.0)	0.854
Pregnancy outcome during follow-up, n (%)				0.593
Live birth^g^	61 (43.3)	38 (41.3)	23 (46.9)	
No live birth	80 (56.7)	54 (58.7)	26 (53.1)	
No pregnancy	70 (87.5)	46 (85.2)	24 (92.3)	
Pregnancy loss	10 (12.5)	8 (14.8)	2 (7.7)	
Median number of reads per sample (IQR)	442 (45–6067)	2167 (532–11,839)	13 (2–45)	

*BMI* body mass index, *IQR* interquartile range.

^a^Seven samples had 0 reads. The clinical characteristics of this group did not differ from the other 42 women who had between 1 and 99 reads, with the exception of smoking.

^b^All continuous variables were compared using the Wilcoxon rank sum test, whereas all categorical variables were compared using the Fisher’s exact test.

^c^Data was missing for one participant in the ≥ 100 reads group.

^d^Data was missing for one participant in the < 100 reads group.

^e^Primary: female has never conceived before. Secondary: female has conceived before.

^f^Other causes of infertility are tubal factor (≥ 100 reads: n = 1, < 100 reads: n = 4), ovulatory disorder (≥ 100 reads: n = 3), endometriosis (≥ 100 reads: n = 1) and mixed causes (≥ 100 reads: n = 3).

^g^One woman conceived spontaneously, and all other women conceived via IVF/ICSI. The sample of the woman who conceived spontaneously had < 100 bacterial reads and was therefore not included in the analyses.

### Sequencing results

Most endometrial samples had low bacterial abundance: 7/141 samples (5.0%) had 0, 42/141 samples (29.8%) had 1–99, and 92/141 samples (65.2%) had ≥ 100 bacterial reads. Participant characteristics and pregnancy outcomes during follow-up were comparable between the women with < 100 and the women with ≥ 100 reads (Table 1). The 92 selected samples with ≥ 100 bacterial reads were selected for all subsequent analyses (Fig. 1). They generated 860,366 reads in total with a median of 2167 reads per sample (interquartile range (IQR) 532 – 11,839) (Table 1). The positive and negative controls did not give cause for concern (“Supplementary Results”, Fig. S1, Table S1). The most abundant genus present in all samples combined was *Lactobacillus* (mean RA 69.3%), followed by *Gardnerella* (mean RA 11.8%) (Fig. S2).

### Endometrial microbiota compositions of women with and without a live birth

**Figure 2:**
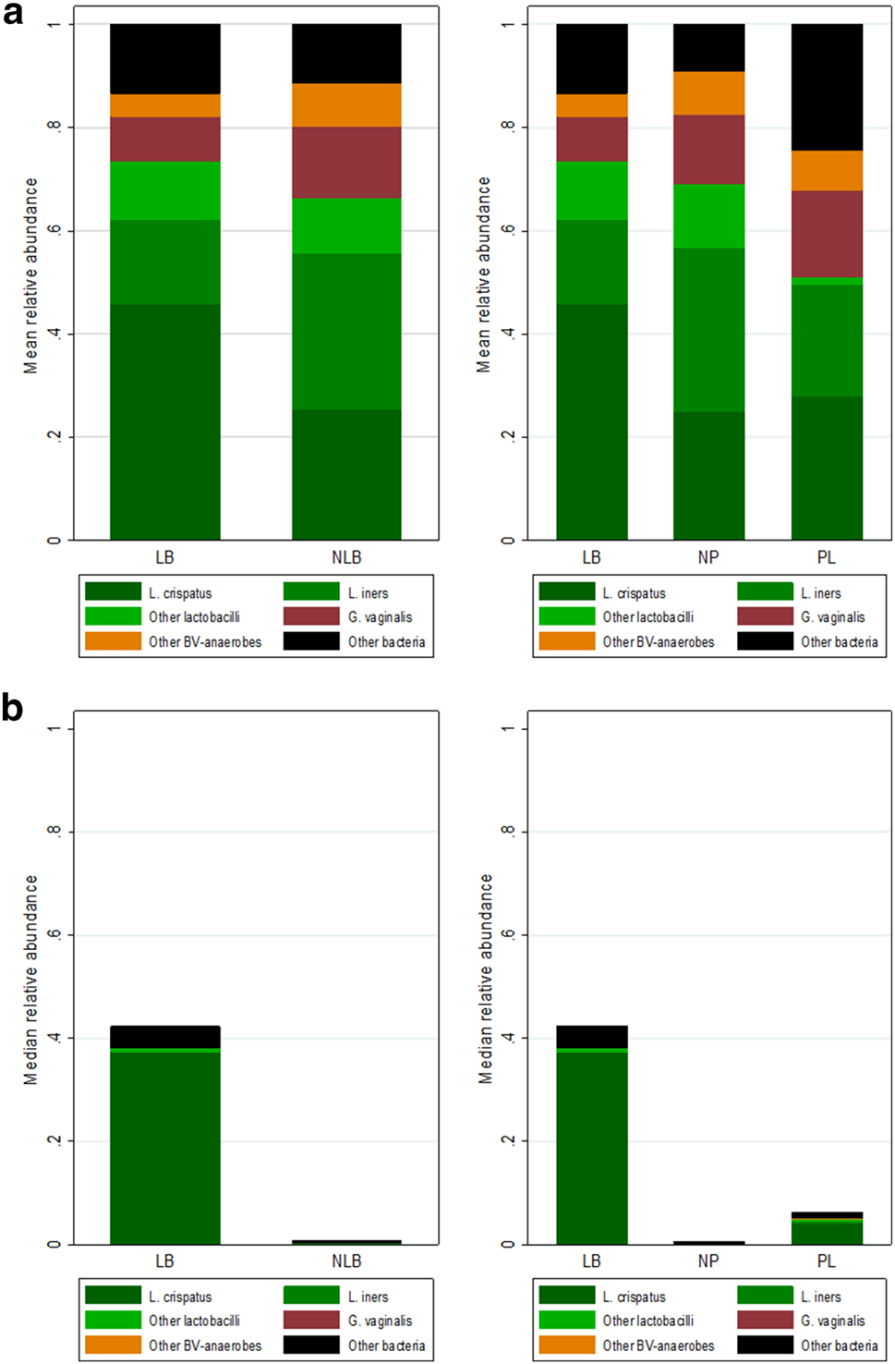
Relative abundance of prespecified bacterial groups by birth outcome. Stacked bar graphs of mean (**a**) and median (**b**) relative abundances of prespecified bacterial groups in the live birth (LB) versus no live birth (NLB) groups. The NLB group was further divided into the no pregnancy (NP) and pregnancy loss (PL) group. BV, bacterial vaginosis; *G. vaginalis*, *Gardnerella vaginalis*; *L. crispatus*, *Lactobacillus crispatus*; *L. iners*, *Lactobacillus iners*.

Among the 92 women with ≥ 100 bacterial reads, 38/92 (41.3%) had a live birth and 54/92 (58.7%) did not. Among the women with no live birth, 46/54 (85.2%) did not get pregnant at all and 8/54 (14.8%) suffered a pregnancy loss. Women with a live birth were significantly younger than women without (median age of 33.5 vs. 36.8 years, respectively; p = 0.016; Table 2). Among the latter group, women with a pregnancy loss had a significantly longer median duration of infertility than women who never conceived (40 vs. 28 months, respectively; p = 0.021). None of the other clinical characteristics differed significantly between the groups. Alpha and beta diversities did not differ between the groups (Table 3, Fig. S3a–d). Untargeted ANCOM-BC analysis did not identify any taxa with significantly different mean RAs between women with and without live birth (Table 3, Fig. S3e), but women who never conceived had significantly higher mean RAs of *Aerococcus* and *Corynebacterium* compared to women with a pregnancy loss (Table 3, Fig. S3f). These mean RAs were, however, only 3% or lower. In targeted Wilcoxon rank sum analyses (comparing prespecified bacterial groups), women with a live birth had a significantly higher *L. crispatus* RA (p = 0.029) and ‘other bacteria’ RA (p = 0.045) than women without a live birth (Table 3, Fig. 2a,b). In addition, a significantly smaller proportion of women with than without a live birth had ≤ 10% *L. crispatus* RA (42.1% and 70.4%, respectively; p = 0.015) (Table 3). For all other prespecified bacterial (sub)groups, the Wilcoxon analysis results and the proportions of women with > 10% RA did not differ between the birth outcome groups (Table 3, Table S2).

**Table 2:** Participants characteristics by birth outcome.

	LB (n = 38)	NLB^a^ (n = 54)			p LB vs. all NLB^b^	p NP vs. PL^b^
		All NLB (n = 54)	NP (n = 46)	PL (n = 8)		
Median female age in years (IQR)	33.5 (28.7–36.3)	36.8 (31.8–39.8)	36.8 (31.6–39.8)	35.8 (32.5–40.6)	**0.016**	0.990
Median female BMI in kg/m^2^ (IQR)^c^	23.0 (21.4–24.9)	24.0 (21.8–26.6)	23.8 (21.8–26.7)	24.9 (21.3–27.0)	0.136	0.951
Median duration of infertility in mo (IQR)	29.0 (22.5–43.5)	30.0 (23.0–43.0)	28.0 (21.8–39.8)	40.0 (31.5–57.3)	0.953	**0.021**
Female smokers, n (%)	3 (7.9)	5 (9.3)	4 (8.7)	1 (12.5)	1.000	0.567
Type of infertility of the female, n (%)^d^					0.835	0.063
Primary	22 (57.9)	30 (55.6)	23 (50.0)	7 (87.5)		
Secondary	16 (42.1)	24 (44.4)	23 (50.0)	1 (12.5)		
Cause of infertility, n (%)					0.188	0.181
Unexplained	15 (39.5)	23 (42.6)	17 (37.0)	6 (75.0)		
Male factor	22 (57.9)	24 (44.4)	22 (47.8)	2 (25.0)		
Other^e^	1 (2.6)	7 (13.0)	7 (15.2)	0		
Median # previous embryo transfers per participant (IQR)	2.0 (1.0–3.0)	1.0 (1.0–3.0)	1.5 (1.0–3.0)	1.0 (1.0–2.0)	0.352	0.322

Significant values are in [bold].

*BMI* body mass index, *IQR* interquartile range, *LB* live birth, *mo* months, *NLB* no live birth, *NP* no pregnancy, *PL* pregnancy loss.

Only samples with ≥ 100 16S rRNA sequencing reads (V1–V2 region) were included.

^a^The NLB participants included participants with a pregnancy loss (PL) as well as participants who did not get pregnant at all (NP).

^b^Continuous variables were compared by Wilcoxon rank sum test and categorical variables by Fisher’s exact test.

^c^Data was missing for one participant in the LB group.

^d^Primary: female has never conceived before. Secondary: female has conceived before.

^e^Other causes of infertility are tubal factor (NLB n = 1), ovulatory disorder (LB n = 1, NLB n = 2), endometriosis (NLB n = 1) and mixed causes (NLB n = 3).

**Table 3:** Endometrial microbiota characteristics by birth outcome.

	LB (n = 38)	NLB^a^ (n = 54)			p LB vs. all NLB^b^	p NP vs. PL^b^
		All NLB (n = 54)	NP (n = 46)	PL (n = 8)		
Alpha diversity						
Mean inverse Simpson diversity (SD)	2.7 (± 1.6)	2.8 (± 2.4)	2.6 (± 2.0)	4.1 (± 3.9)	0.654	0.154
Mean Chao1 diversity (SD)	15.9 (± 26.7)	13.5 (± 15.9)	12.7 (± 16.2)	18.0 (± 14.0)	0.769	0.073
Untargeted ANCOM-BC results^b,c^						
Mean RA *Aerococcus* genus (SD)	0.2 (± 1.2)	0.1 (± 0.5)	0.2 (± 0.5)	0	NS	**< 0.001**
Mean RA *Corynebacterium* genus (SD)	3.3 (± 11.5)	1.9 (± 6.0)	2.0 (± 6.5)	1.0 (± 1.7)	NS	**< 0.001**
Targeted bacterial groups (specified a priori)^b,c^						
* Lactobacillus* genus					0.893	0.450
Median RA (IQR)	94.2 (39.1–99.2)	94.5 (18.9–99.6)	95.8 (32.5–99.7)	48.1 (7.6–98.4)		
95% CI	80.1–97.7	69.9–98.8	69.9–99.3	1.5–100.0		
Mean RA (SD)	73.5 (± 35.2)	66.4 (± 41.5)	69.0 (± 40.6)	51.0 (± 46.5)		
95% CI	61.2–77.4	55.0–77.7	57.0–81.1	12.2–89.9		
* L. crispatus*					**0.029**	0.510
Median RA (IQR)	37.4 (0–94.4)	0 (0–55.7)	0 (0–55.7)	4.2 (0–37.8)		
95% CI	0–91.5	0–0	0–0	0–99.5		
Mean RA (SD)	45.8 (± 44.3)	25.4 (± 41.4)	25.0 (± 41.4)	27.9 (± 44.1)		
95% CI	31.2–60.4	14.1–36.7	12.7–37.3	0–64.8		
* L. iners*					0.141	0.971
Median RA (IQR)	0 (0–15.9)	0.3 (0–71.7)	0.2 (0–71.7)	0.3 (0–22.3)		
95% CI	0–5.5	0–21.5	0–63.1	0–92.8		
Mean RA (SD)	16.5 (± 30.6)	30.2 (± 40.5)	31.6 (± 41.0)	21.7 (± 39.0)		
95% CI	6.4–26.5	19.1–41.2	19.5–43.8	0–54.3		
Other lactobacilli					0.203	0.495
Median RA (IQR)	0.7 (0–5.0)	0 (0–4.4)	0 (0–6.8)	0.5 (0.2–1.4)		
95% CI	0–2.6	0–0.6	0–0.6	0–6.6		
Mean RA (SD)	11.2 (± 24.5)	10.8 (± 27.1)	12.4 (± 29.0)	1.3 (± 2.2)		
95% CI	3.1–19.3	3.4–18.1	3.8–21.0	0–3.2		
* Gardnerella* genus					0.541	1.000
Median RA (IQR)	0 (0–0)	0 (0–1.6)	0 (0–1.6)	0 (0–9.3)		
95% CI	0–0	0–0	0–0	0–98.3		
Mean RA (SD)	8.6 (± 20.3)	14.0 (± 28.7)	13.4 (± 27.8)	16.9 (± 35.4)		
95% CI	2.0–15.3	6.1–21.8	5.2–21.7	0–46.5		
Other BV-anaerobes					0.558	0.802
Median RA (IQR)	0 (0–1.5)	0 (0–2.6)	0 (0–4.5)	0.1 (0–0.6)		
95% CI	0–2.4	0–1.4	0–0.6	0–58.6		
Mean RA (SD)	4.4 (± 10.8)	8.3 (± 18.8)	8.4 (± 18.7)	7.6 (± 20.6)		
95% CI	0.9–8.0	3.2–13.4	2.9–14.0	0–24.8		
Other bacteria^d^					**0.045**	0.233
Median RA (IQR)	4.7 (0.3–13.2)	0.5 (0–8.9)	0.4 (0–5.9)	1.2 (0.1–36.8)		
95% CI	0.8–6.9	0.1–1.4	0–1.4	0–91.4		
Mean RA (SD)	13.5 (± 23.7)	11.4 (± 22.8)	9.1 (± 18.6)	24.5 (± 38.5)		
95% CI	5.7–21.2	5.2–17.6	3.6–14.6	0–56.6		
* L. crispatus* RA subgroups, n (%)					**0.015**	0.679
≤ 10	16 (42.1)	38 (70.4)	33 (71.7)	5 (62.5)		
11–89	9 (23.7)	4 (7.4)	3 (6.5)	1 (12.5)		
≥ 90	13 (34.2)	12 (22.2)	10 (21.7)	2 (25.0)		

Significant values are in [bold].

*BV* bacterial vaginosis, *CI* confidence interval, *IQR* interquartile range, *LB* live birth, *mo* months, *NLB* no live birth, *NP* no pregnancy, *NS* not significant, *PL* pregnancy loss, *RA* relative abundance, *SD* standard deviation.

Only samples with ≥ 100 16S rRNA sequencing reads (V1–V2 region) were included.

^a^The NLB participants included participants with a pregnancy loss (PL) as well as participants who did not get pregnant at all (NP).

^b^ANCOM-BC analyses were corrected for multiple testing and were used to identify individual taxa with significantly different RAs in comparison groups in an untargeted manner. In the targeted analyses (using prespecified bacterial groups and subgroups), we assumed that the data were not normally distributed. All p-values were calculated by Wilcoxon rank sum tests only, but we are showing both median and mean RAs for illustrative purposes.

^c^Relative abundances are presented as percentages of the number of reads of the taxon out of the total number of bacterial reads.

^d^The group of “other bacteria” contains skin bacteria, unresolved bacteria and minority taxa that could not be assigned to any of the other categories.

### Endometrial microbiota compositions by infertility causes

Male factor infertility was most common (46/92; 50.0%), followed by unexplained infertility (38/92; 41.3%), and other causes (8/92; 8.7%). Women with unexplained and other causes of infertility were significantly older than women with male factor infertility (median ages of 37.7, 37.7, and 32.7 years, respectively), and also had a longer median duration of infertility (median infertility duration of 35.0, 37.0 and 27.5 months, respectively; Table S3). None of the other clinical characteristics differed significantly between the groups. Endometrial microbiota profiles did not differ in alpha and beta diversities (Table S4, Fig. S4a,b). Untargeted ANCOM-BC analysis and targeted Wilcoxon rank sum analyses did not identify any taxa or prespecified bacterial groups with significantly different RAs between the infertility causes groups (Table S4, Fig. S4c–e). However, the proportion of women with ≤ 10% *L. crispatus* was significantly lower in the male factor infertility group compared to in the other two groups (p = 0.030) (Table S4). For all other prespecified bacterial (sub)groups the proportions of women with > 10% RA, did not differ between the infertility causes groups (Table S4).

### Endometrial microbiota compositions by primary vs. secondary infertility

Primary infertility was present in 52/92 (56.5%) women and secondary infertility in 40/92 (43.5%) women. Women with secondary infertility were significantly older than women with primary infertility (median age of 36.8 vs. 33.4 years, respectively; p = 0.001) (Table S5). The other clinical characteristics did not differ significantly between the two groups (Table S5). Alpha and beta diversities did not differ significantly either (Table S6, Fig. S5a,b). Untargeted ANCOM-BC analysis identified a significantly lower mean *Gardnerella* genus RA in women with primary compared to women with secondary infertility (6.1% vs. 19.2%, respectively; p = 0.030) (Table S6, Fig. S5c). In targeted Wilcoxon rank sum analyses, women with primary compared to secondary infertility had a significantly higher *L. crispatus* RA (p = 0.004), and a trend towards lower *G. vaginalis* RA (p = 0.051) (Table S6, Fig. S5d,e). The proportions of women with ≤ 10% *L. crispatus* and > 10% *G. vaginalis* also differed significantly between the groups (p = 0.009 and 0.019 respectively), with women with primary infertility being more likely to have higher levels of *L. crispatus* and lower levels of *G. vaginalis* than women with secondary infertility (Table S6).

## Discussion

*Lactobacillus* was the most abundant genus in the majority of samples. While untargeted analysis did not identify any differentially relatively abundant taxa by birth outcomes, women with a live birth had significantly higher *L. crispatus* RA than women with no live birth in targeted analysis. Although untargeted and targeted analyses did not identify any differentially relatively abundant taxa between women with different infertility causes, we revealed that women with male factor infertility were significantly less likely to have endometrial microbiota containing ≤ 10% *L. crispatus*. We observed the largest differences in endometrial microbiota profiles between women with primary or secondary infertility, with the latter women showing several signs of endometrial microbiota dysbiosis (i.e. reduced *L. crispatus* and increased BV-anaerobes, particularly *G. vaginalis*).

*Lactobacillus* was the most relatively abundant genus in almost all endometrium samples in our study, which is consistent with several previous studies^3,5–8,28–30^. However, one previous study using endometrial tissue, obtained after hysterectomy, detected hardly any lactobacilli at all^31^. It is well-known that lactobacilli are by far the most common bacteria in the vagina and cervix, except in BV patients and in women with other less common types of vaginal dysbiosis^2,32^. Cervicovaginal bacteria can travel through the endocervical canal^33^. It would therefore seem unlikely for the endometrium to not contain lactobacilli. However, endometrial sample contamination may also explain their presence. When the endometrial sample is collected via the endocervical canal, the clinician might pick-up cervicovaginal bacteria during the sampling procedure. We were very much aware of this risk and tried to minimize such contamination (see “Methods”). We believe that the endometrium likely does contain lactobacilli in most women and offer an alternative explanation as to why Winters et al. may not have detected them^31^. While they did use endometrial tissue obtained after hysterectomy, as opposed to a sample through the endocervical canal (as we and others did), their study population consisted of 25 women whose endometrial tissue was abnormal due to fibroids or endometrial hyperplasia. Bacteria metabolize carbohydrates they obtain from tissues and mucus^34^, and altered endometrial tissue may therefore also alter the endometrial microbiota composition^35^.

Contamination may also be problematic for a second reason. The endometrium typically contains few micro-organisms. The introduction of small quantities of contaminants (for example, via reagents) might have a large impact on the sequencing results, making it difficult to identify true biological signals^36^. We therefore included controls in our DNA extraction and sequencing pipelines and did not detect any such contaminants.

We showed a beneficial association of endometrial *L. crispatus* with ART outcome, which is in agreement with findings by others who assessed cervicovaginal^37–42^ or endometrial microbiota compositions^8^ in relation to birth outcomes. While evidence for this beneficial association of *L. crispatus* is mounting, the clinical significance remains unclear. The ability to conceive, and the success of ART treatment, depends on many factors, of which the presence of specific bacteria and/or inflammation in the female genital tract is only one^43,44^. At the moment, it is still unclear whether optimizing the female genital tract microbiota might improve the ART success rate, and if yes, to what extent. Our own data shows this heterogeneity quite clearly: while women who conceived had a ‘healthier’ microbiota on average than women who did not conceive, not all *L. crispatus*-dominated women did conceive. Even if *L. crispatus*-domination is considered desirable, the next question is how to achieve it. A recent systematic review of lactobacilli-containing vaginal probiotics showed that the concept is promising, but currently available products require improvement, especially in terms of their ability to colonize the female genital tract^45^. To date, two Japanese studies have investigated endometrial microbiota manipulation as a means to improve ART success^46,47^. One study investigated combinations of oral and vaginal lactobacilli-containing probiotics and metronidazole^46^, while the other investigated oral lactoferrin supplementation as a prebiotic^47^. However, the results of these studies are difficult to interpret due to lack of no-intervention control groups. Once pre- or probiotics capable of increasing the colonization of lactobacilli in the female genital tract have been developed, randomised controlled clinical trials would have to be conducted to determine their effect on ART outcomes empirically.

Our data support our hypothesis that women with male factor infertility would have ‘healthier’ endometrial microbiota than women with unexplained or other causes of infertility. Other than the differences in *L. crispatus* RA, we did not find any evidence for severe female genital tract dysbiosis (e.g. high RA of BV-anaerobes or other bacteria) as a potential explanation for infertility in the total group of primary or secondary unexplained infertility cases. However, our data suggest that severe dysbiosis may be associated with secondary infertility. To our knowledge, this has not been described before. It should be noted that women with secondary infertility were also on average 3–4 years older than women with primary infertility. While age could act as a confounder, we would not normally expect an age-related decrease of lactobacilli in the female genital tract until menopause^48,49^. A recent study demonstrated that the vaginal and uterine microbiota composition remained stable with increasing age among women younger than 40 years^50^.

Our study is unique in that we could prospectively obtain endometrial biopsies as part of an intervention trial. Endometrial sampling is invasive and data on endometrial microbiota are therefore scarce. While our sample size was sufficient for detecting some important microbiota profile differences between our comparison groups, an even larger sample size would have been desirable to better accommodate the large variability in infertility causes and microbiota profiles and adjust for confounding. In addition, we did not have data on a few important potential confounders such as ethnicity, recent use of antibiotics or immunosuppressant drugs, and history of vaginitis, cervicitis, endometritis, pelvic inflammatory disease, and uterine surgery. As already noted, we tried to minimize cervicovaginal contamination during transcervical sampling, as well as contamination from other sources, by promoting strict sampling and hygiene procedures. We also included positive and negative (blank) controls in our sequencing pipeline to assess the presence of contaminant DNA. However, we cannot be sure that contamination was ruled out. Related to this, we had to exclude one-third of the samples that we sequenced, because they had fewer than 100 bacterial reads. Another limitation was that 16S rRNA gene sequencing only allows inferences to be made about bacteria, and not about other micro-organisms that are known to be common in the female reproductive tract (such as viruses and yeasts), which may also play a role in ART outcome^51^.

## Conclusion

In summary, our data point towards a beneficial association of *L. crispatus* with IVF/ICSI success, and suggest that secondary infertility may be associated with endometrial dysbiosis more often than primary infertility. These hypotheses should be tested in rigorous well-powered longitudinal studies. The clinical implications of our study are unclear. Further research is needed to investigate mechanisms linking the endometrial microbiota to the process of implantation^52,53^, as well as clinical trials of potential interventions.

## Supplementary Information

Supplementary Information 1.

Supplementary Information 2.

## Data Availability

Sequencing data has been made available on the European Nucleotide Archive under project code PRJEB53740 (https://www.ebi.ac.uk/ena/browser/view/PRJEB53740). R scripts are available on https://gitlab.com/PB_Stege/diet_microbiome_resistome/.

## Abbreviations

16S rRNA: 16S ribosomal ribonucleic acid
ANCOM-BC: Analysis of compositions of microbiomes with bias correction
ART: Assisted reproductive technology
BV: Bacterial vaginosis
Clr: Centered log ratio
DNA: Deoxyribonucleic acid
FDR: False discovery rate
ICSI: Intracytoplasmic sperm injection
IQR: Interquartile range
IVF: In vitro fertilization
LB: Live birth
NGS: Next generation sequencing
NLB: No live birth
PCA: Principal component analysis
RA: Relative abundance
WHO: World Health Organization
