# Supplementary material for: The endometrial microbiota of women with or without a live birth within 12 months after a first failed IVF/ICSI cycle
Source: Sci Rep. 2023 Mar 1;13:3444. doi: 10.1038/s41598-023-30591-2 (PMC9977966; doi:10.1038/s41598-023-30591-2)

Supplement 1

Title: The endometrial microbiota of women with or without a live birth within 12 months after a first failed IVF/ICSI cycle

Authors: Bich Ngoc Bui; Nienke van Hoogenhuijze; Marco Viveen; Femke Mol; Gijs Teklenburg; Jan-Peter de Bruin; Dagmar Besselink; Linda Stevens Brentjens; Shari Mackens; Malbert R.C. Rogers; Gaby Steba; Frank Broekmans; Fernanda L. Paganelli; Janneke H.H.M. van de Wijgert

Supplementary Methods

Total DNA isolation

One-third of the snap-frozen endometrial tissue of each participant was thawed. Half of it was used for the microbiota analysis described in this study. Stainless steel beads (Ø 5 mm, Qiagen, Germany) and 50 µl nuclease-free water were added to the tissue, after which the tissue was homogenized using the TissueLyser II system (Qiagen, Germany). To each sample, 800 µl RLT Plus lysis buffer with β-mercaptoethanol was added, followed by overnight incubation, while shaking at 60°C. Subsequently, 500 µl zarconium 0.1 mm beads were added and beat beating was applied 2 times at 3.5 m/s for 2 min (Mini-Beadbeater-96, Biospec products), followed by 2 min incubation on ice interval. Total DNA was extracted using the Qiagen Allprep DNA/RNA Mini Kit following the manufacturer's protocol. Two different positive control mock communities (ZymoBIOMICS Microbial Community Standard (D6300) and Standard II (Log Distribution) (D6310)) as well as negative DNA extraction controls (Microbial DNA-free water (Qiagen art nr. 338132)) were included in all steps of the analysis. No template controls (NTC) (Microbial DNA-free water (Qiagen art nr. 338132)) were included in the PCR steps.

16S rRNA sequencing and bioinformatics

16S rRNA sequencing was done according to Fadrosh et al. (1) but primers were modified to target and amplify the V1-V2 region of the 16S rRNA gene: forward primer 5' – AGM GTT YGA

TYM TGG CTC AG – 3′ and reverse primer 5′ – GCT GCC TCC CGT AGG AGT – 3′ (2,3). The V1-V2 region was chosen to minimise human DNA amplification (4). Sequencing was performed on a MiSeq platform (Illumina, USA), using the MiSeq Reagent Kit v3 (Illumina, USA). The QIIME2 microbial community analysis pipeline (version 2018.8) (5) was used with DADA2 (6) for amplicon sequence variant detection, and SILVA as the 16S rRNA reference gene database (SILVA 138) (7). Reads classified as human DNA, mitochondria or 'unassigned' were removed. All data from samples with fewer than 100 bacterial reads were discarded.

Supplementary Results

Sequencing results positive and negative controls

The positive mock community controls (n=9) had a total of 728,660 bacterial reads with a median of 98,329 reads per sample (IQR 52,347 – 112,266). The relative abundances of the Standard Mock Community (D6300) was as expected, with approximately equal distribution of 8 bacterial taxa (**Figure S1**). In addition, a log distribution with a detection limit of 0.089% was observed in the Standard Mock Community II (D6310) (**Figure S1**). The theoretical microbial composition of the standards can be found in the Zymo Research Instruction Manuals of the corresponding standards (see (8) for D6300 and (9) for D6310). The negative controls (n=8) had a total of 157 bacterial reads with a median of 0 reads per sample (IQR 0 – 5). The no template controls (NTC) (n=24) had a total of 378 reads with a median of 2 reads per sample (IQR 0 – 18). One negative control had 146 reads, however, showing high abundance of *Occallatibacter* and *Meiothermus* (**Table S1**). *Occallatibacter* was not present in the study samples, whereas *Meiothermus* was only present in three study samples with very low reads (<10). All other negative controls (n=7) and all NTC (n=24) contained fewer than 100 reads (**Table S1**).

References

1. Fadrosh DW, Ma B, Gajer P, Sengamalay N, Ott S, Brotman RM, et al. An improved dual-indexing approach for multiplexed 16S rRNA gene sequencing on the Illumina MiSeq platform. Microbiome. 2014;2(1):1–7.

2. Browne HP, Forster SC, Anonye BO, Kumar N, Neville BA, Stares MD, et al. Culturing of "unculturable" human microbiota reveals novel taxa and extensive sporulation. Nature. 2016;533(7604):543–6.

3. Elliott DRF, Walker AW, O'Donovan M, Parkhill J, Fitzgerald RC. A non-endoscopic device to sample the oesophageal microbiota: a case-control study. Lancet Gastroenterol Hepatol [Internet]. 2017;2(1):32–42. Available from: http://dx.doi.org/10.1016/S2468-1253(16)30086-3

4. Walker SP, Barrett M, Hogan G, Flores Bueso Y, Claesson MJ, Tangney M. Non-specific amplification of human DNA is a major challenge for 16S rRNA gene sequence analysis. Sci Rep [Internet]. 2020;10(1):1–7. Available from: https://doi.org/10.1038/s41598-020-73403-7

5. Bolyen E, Rideout JR, Dillon MR, Bokulich NA, Abnet CC, Al-Ghalith GA, et al. Reproducible, interactive, scalable and extensible microbiome data science using QIIME 2. Nat Biotechnol. 2019;37(8):852–7.

6. Callahan BJ, McMurdie PJ, Rosen MJ, Han AW, Johnson AJA, Holmes SP. DADA2: High-resolution sample inference from Illumina amplicon data. Nat Methods. 2016;13(7):581–3.

7. Quast C, Pruesse E, Yilmaz P, Gerken J, Schweer T, Yarza P, et al. The SILVA ribosomal RNA gene database project: Improved data processing and web-based tools. Nucleic Acids Res. 2013;41(D1):590–6.

8. ZymoResearch. Instruction Manual: ZymoBIOMICS™ Microbial Community Standard D6300 [Internet]. Available from: https://files.zymoresearch.com/protocols/_d6300_zymobiomics_microbial_community_standard.pdf

9. ZymoResearch. Instruction Manual: ZymoBIOMICS™ Microbial Community Standard II (Log Distribution) D6310 [Internet]. Available from: https://files.zymoresearch.com/protocols/_d6310_zymobiomics_microbial_community_standard_ii_(log_distribution).pdf

Supplementary Tables and Figures

Figure S1: Positive controls. Relative abundances of bacterial taxa in the mock communities that were used as positive controls in this study. In the left panel, the average distribution of 8 bacterial taxa in the ZymoBIOMICS Microbial Community Standard II (Log Distribution) (D6310) (n=4) is displayed and in the right panel the average distribution of 8 bacterial taxa in the ZymoBIOMICS Microbial Community Standard (D6300) (n=4 samples). See **Table S1** for sequencing results of the negative control samples.

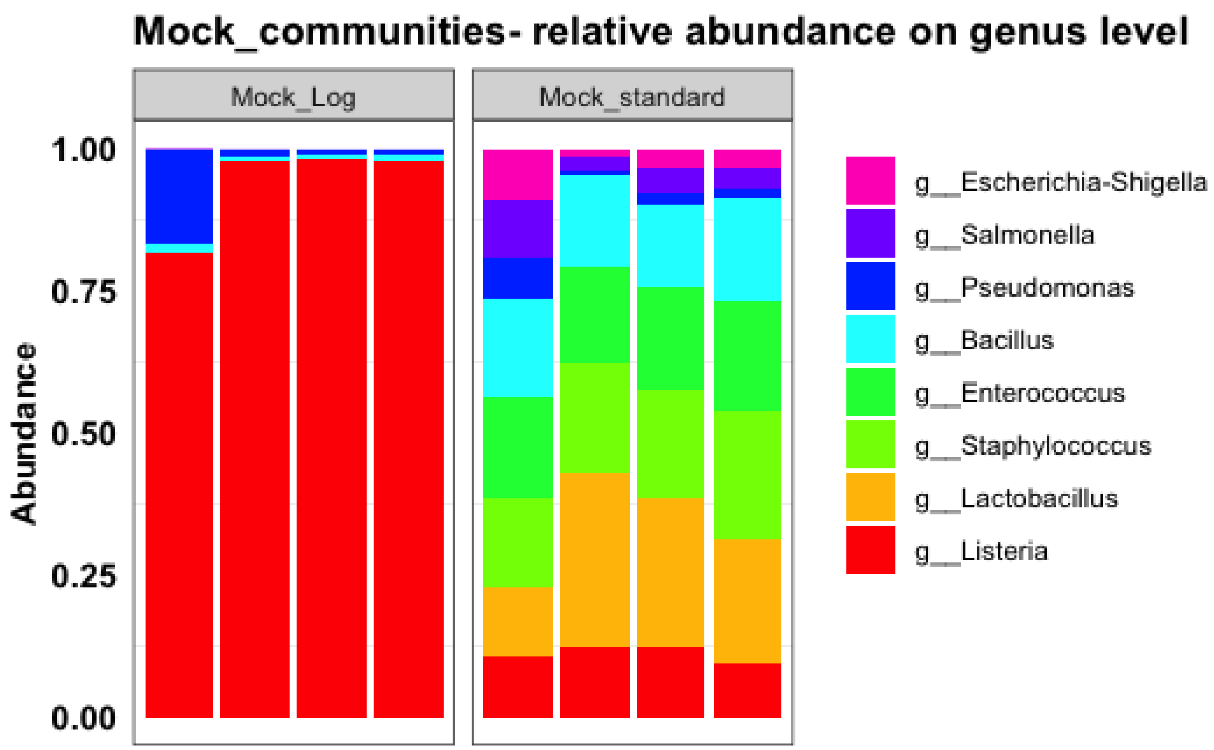

Table S1: Sequencing results of negative control samples. Number of bacterial reads in negative controls (sterile water DNA extraction) and no template controls (NTC; sterile water). Cells containing bacterial reads are highlighted in red.

Sample	Total number of bacterial reads	Lactobacillus	Listeria	Staphylococcus	Escherichia-Shigella	Corynebacterium	Cutibacterium	Dyella	Streptococcus	Paracoccus	Meiothermus	Haemophilus	Ralstonia	Bradyrhizobium	Occallatibacter	Geitlerinema	Flectobacillus	Enhydrobacter	Brachybacterium	Clostridia_UCG-014	Fimbriimonadaceae	Dorea	Alloprevotella
Negative control 1	7	0	0	0	0	0	0	0	0	0	0	0	0	0	0	0	7	0	0	0	0	0	0
Negative control 2	0	0	0	0	0	0	0	0	0	0	0	0	0	0	0	0	0	0	0	0	0	0	0
Negative control 3	0	0	0	0	0	0	0	0	0	0	0	0	0	0	0	0	0	0	0	0	0	0	0
Negative control 4	4	0	0	0	0	0	0	0	0	0	0	0	0	0	0	0	0	0	0	0	4	0	0
Negative control 5	146	0	0	14	0	0	0	7	0	0	85	0	0	0	40	0	0	0	0	0	0	0	0
Negative control 6	0	0	0	0	0	0	0	0	0	0	0	0	0	0	0	0	0	0	0	0	0	0	0
Negative control 7	0	0	0	0	0	0	0	0	0	0	0	0	0	0	0	0	0	0	0	0	0	0	0
Negative control 8	0	0	0	0	0	0	0	0	0	0	0	0	0	0	0	0	0	0	0	0	0	0	0
NTC 1	0	0	0	0	0	0	0	0	0	0	0	0	0	0	0	0	0	0	0	0	0	0	0
NTC 2	0	0	0	0	0	0	0	0	0	0	0	0	0	0	0	0	0	0	0	0	0	0	0
NTC 3	1	0	0	0	0	0	0	0	1	0	0	0	0	0	0	0	0	0	0	0	0	0	0
NTC 4	50	0	0	0	0	10	0	0	0	0	0	0	0	0	0	40	0	0	0	0	0	0	0
NTC 5	10	0	0	0	0	0	0	0	0	10	0	0	0	0	0	0	0	0	0	0	0	0	0
NTC 6	0	0	0	0	0	0	0	0	0	0	0	0	0	0	0	0	0	0	0	0	0	0	0
NTC 7	0	0	0	0	0	0	0	0	0	0	0	0	0	0	0	0	0	0	0	0	0	0	0
NTC 8	0																						
NTC 9	18	0	0	0	4	0	0	0	14	0	0	0	0	0	0	0	0	0	0	0	0	0	0
NTC 10	89	0	0	0	0	0	0	0	0	0	0	89	0	0	0	0	0	0	0	0	0	0	0
NTC 11	49	0	0	0	0	0	10	0	0	0	0	0	39	0	0	0	0	0	0	0	0	0	0
NTC 12	0	0	0	0	0	0	0	0	0	0	0	0	0	0	0	0	0	0	0	0	0	0	0
NTC 13	16	0	0	0	0	0	0	0	16	0	0	0	0	0	0	0	0	0	0	0	0	0	0
NTC 14	19	0	0	0	0	0	0	0	0	0	0	0	0	19	0	0	0	0	0	0	0	0	0
NTC 15	22	0	0	0	0	0	0	0	0	0	0	0	0	0	0	0	0	0	22	0	0	0	0
NTC 16	0	0	0	0	0	0	0	0	0	0	0	0	0	0	0	0	0	0	0	0	0	0	0
NTC 17	1	0	1	0	0	0	0	0	0	0	0	0	0	0	0	0	0	0	0	0	0	0	0
NTC 18	0																						
NTC 19	0	0	0	0	0	0	0	0	0	0	0	0	0	0	0	0	0	0	0	0	0	0	0
NTC 20	0	0	0	0	0	0	0	0	0	0	0	0	0	0	0	0	0	0	0	0	0	0	0
NTC 21	86	0	0	12	0	42	0	0	0	0	0	0	0	0	0	0	0	15	0	5	0	4	0
NTC 22	3	0	0	0	0	0	0	0	0	0	0	0	0	0	0	0	0	0	0	0	0	0	3
NTC 23	3	3	0	0	0	0	0	0	0	0	0	0	0	0	0	0	0	0	0	0	0	0	0
NTC 24	11	0	0	11	0	0	0	0	0	0	0	0	0	0	0	0	0	0	0	0	0	0	0

Figure S2: Heatmap of all included samples. Heatmap of the 20 most relatively abundant bacterial genera in all 92 endometrium samples with ≥100 bacterial reads that were included in the analysis. The heatmap was generated with the phyloseq and microbiome packages in R studio (v4.1.0).

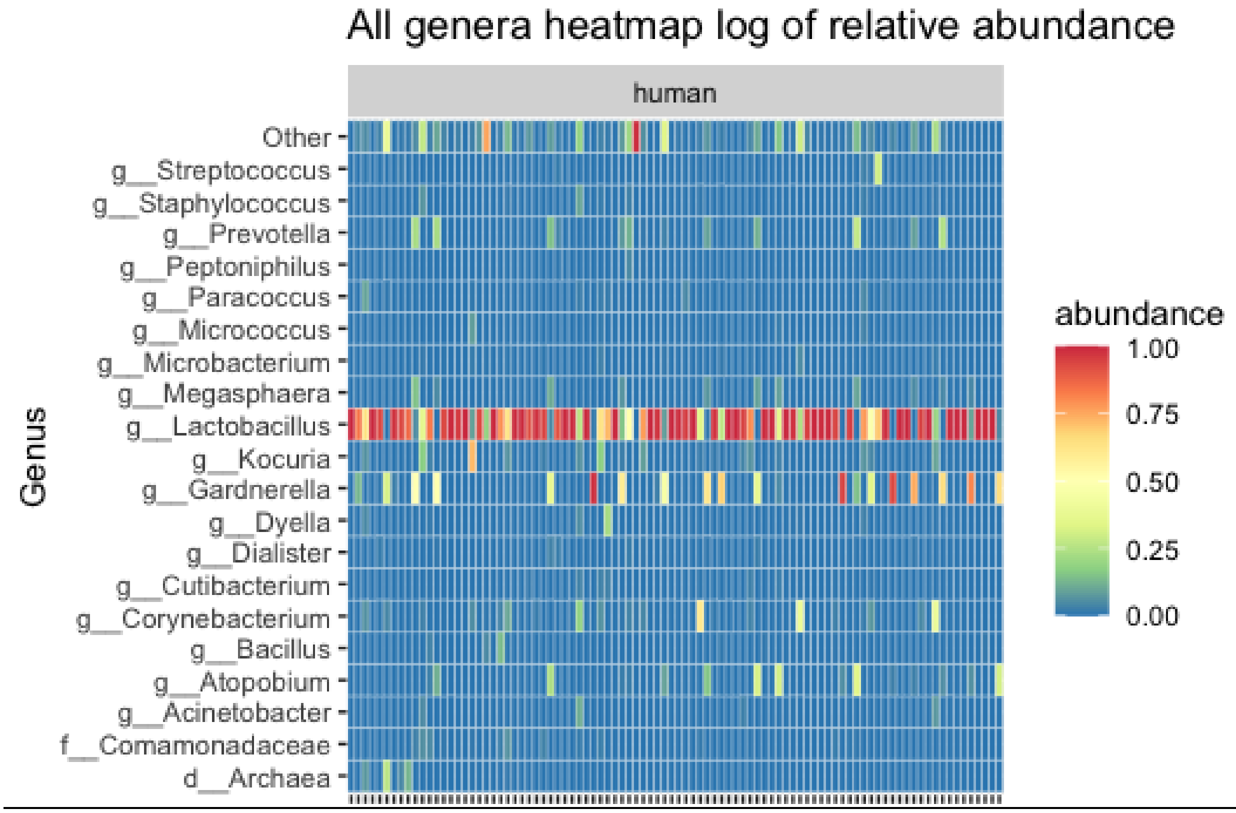

Figure S3: Endometrial microbiota compositions by birth outcomes. Comparison of endometrial microbiota composition and diversity between the live birth (LB), no live birth (NLB), no pregnancy (NP) and pregnancy loss (PL) groups. **a,c.** Graphical visualisation of alpha diversity analysis of LB vs NLB (**a**) and NP vs PL (**c**). Boxplots represent median alpha diversity by Chao1 index and Inverse Simpson index with interquartile range. **b,d.** Graphical visualisation of beta diversity analysis by principal component analysis (PCA) of LB vs NLB (**b**) and NP vs PL (**d**), based on centered log-ratio (clr) transformation of the relative abundances of genera. **e,f.** Heatmaps representing the 20 most relatively abundant bacterial genera in endometrial samples of LB vs NLB (**e**), and NP vs PL (**f**). Each column represents a subject, whereas each row represents a genus. The heatmaps were generated with the phyloseq and microbiome packages in R studio (v4.1.0).

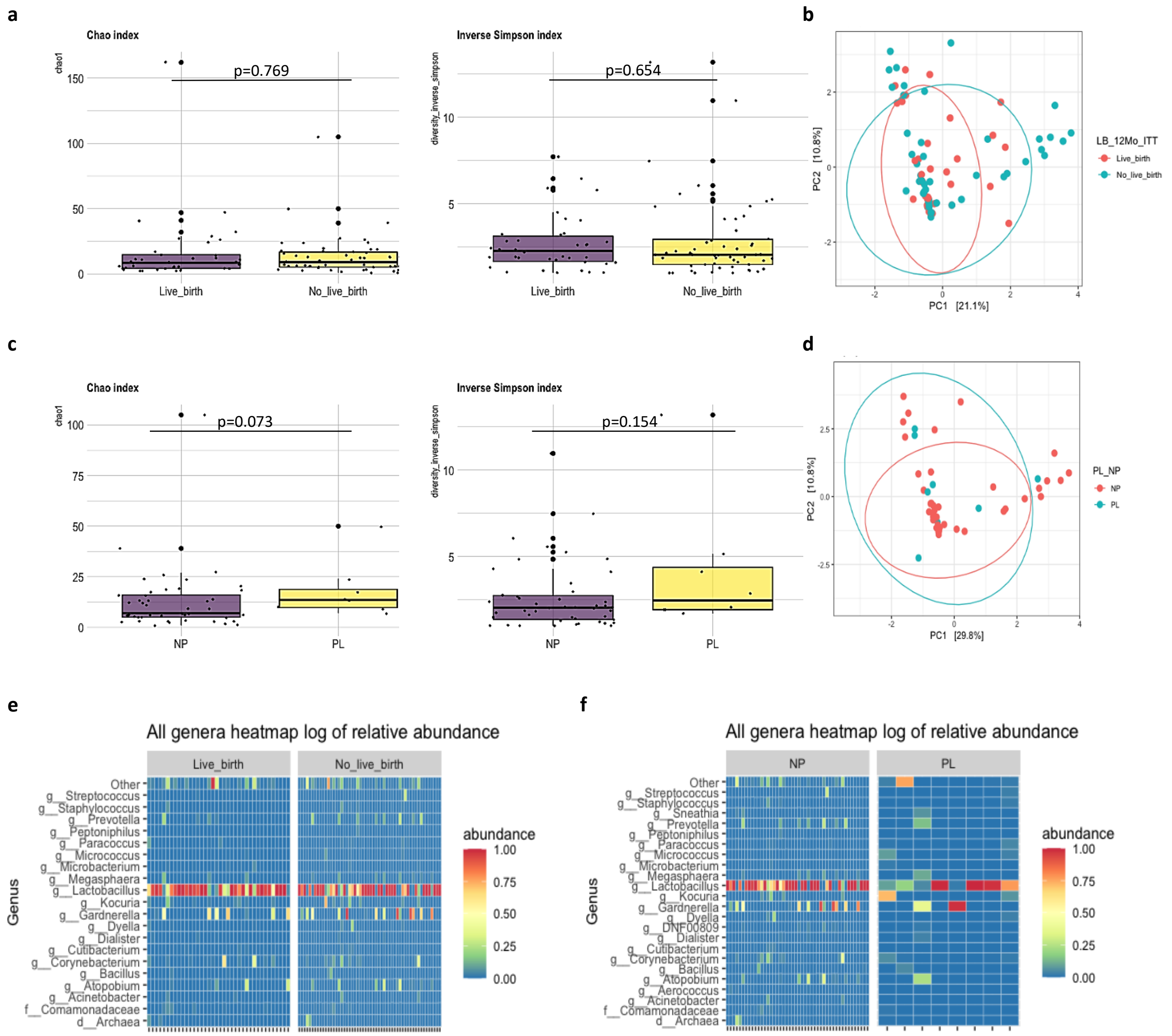

Table S2. Endometrial microbiota characteristics by birth outcome[a]. For additional characteristics, see Table 3 in the main manuscript

	LB (n=38)	NLB[b] (n=54) All NLB (n=54)	NP (n=46)	PL (n=8)	p LB vs. all NLB[c]	p NP vs. PL[c]
Lactobacillus genus RA, n (%)					0.273	0.542
≤10%	4 (10.5)	13 (24.1)	10 (21.7)	3 (37.5)		
11-89%	10 (26.3)	13 (24.1)	11 (23.9)	2 (25.0)		
≥90%	24 (63.2)	28 (51.9)	25 (54.3)	3 (37.5)		
L. iners RA, n (%)					0.782	0.858
≤10%	26 (68.4)	33 (61.1)	27 (58.7)	6 (75.0)		
11-89%	8 (21.2)	13 (24.1)	12 (26.1)	1 (12.5)		
≥90%	4 (10.5)	8 (14.8)	7 (15.2)	1 (12.5)		
Other lactobacilli RA, n (%)					0.838	0.738
≤10%	30 (78.9)	45 (83.3)	37 (80.4)	8 (100)		
11-89%	6 (11.1)	6 (11.1)	6 (13.0)	0		
≥90%	2 (5.3)	3 (5.6)	3 (6.5)	0		
>10% *Gardnerella* genus RA, n (%)	6 (15.8)	13 (24.1)	11 (23.9)	2 (25.0)	0.436	1.000
>10% Other BV-anaerobes RA, n (%)	5 (13.2)	9 (16.7)	8 (17.4)	1 (12.5)	0.772	1.000
>10% Other bacteria RA, n (%)[d]	11 (28.9)	13 (24.1)	10 (21.7)	3 (37.5)	0.636	0.382
>10% BV-anaerobes or other bacteria RA, n (%)	13 (34.2)	21 (38.9)	17 (37.0)	4 (50.0)	0.668	0.697

BV, bacterial vaginosis; LB, live birth; NLB, no live birth; NP, no pregnancy; PL, pregnancy loss; RA, relative abundance.

[a]Only samples with ≥100 16S rRNA sequencing reads (V1-V2 region) were included.

[b]The NLB participants included participants with a pregnancy loss (PL) as well as participants who did not get pregnant at all (NP).

[c]Statistical analysis was performed using the Fisher's exact test.

[d]The group of "other bacteria" contains skin bacteria, unresolved bacteria and minority taxa that could not be assigned to any of the other categories.

Table S3. Participant characteristics by infertility causes[a]

	Male factor (n=46)	Unexplained infertility (n=38)	Other infertility[b] (n=8)	p male factor vs. unexplained[c]	p male factor vs. unexplained + other[c]	p unexplained vs. male factor + other[c]
Median female age in years (IQR)	32.7 (28.9-36.2)	37.7 (33.9-40.6)	37.7 (30.6-40.6)	**<0.001**	**<0.001**	**0.001**
Median female BMI in kg/m^2 (IQR)[d]	23.0 (21.5-25.8)	23.8 (21.9-25.3)	24.4 (22.0-27.8)	0.625	0.439	0.831
Median infertility duration in mo (IQR)	27.5 (21.0-36.0)	35.0 (24.8-51.0)	37.0 (18.0-71.8)	**0.019**	**0.026**	**0.034**
Female smokers, n (%)	6 (13.0)	2 (5.3)	0	0.284	0.267	0.463
Type of infertility of the female, n (%)[e]				0.187	0.141	0.393
Primary	30 (65.2)	19 (50.0)	3 (37.5)			
Secondary	16 (37.2)	19 (50.0)	5 (62.5)			
Median # previous embryo transfers per participant (IQR)	1.5 (1.0-3.3)	2.0 (1.0-3.0)	2.0 (1.0-3.0)	0.885	0.957	0.816
Pregnancy outcome during follow-up				0.511	0.290	0.832
Live birth	22 (47.8)	15 (39.5)	1 (12.5)			
No live birth	24 (52.2)	23 (60.5)	7 (87.5)			
No pregnancy	22 (91.7)	17 (73.9)	7 (100)			
Pregnancy loss	2 (8.3)	6 (26.1)	0			

BMI, body mass index; IQR, interquartile range.

[a]Only samples with ≥100 16S rRNA sequencing reads (V1-V2 region) were included.

[b]Other causes of infertility are tubal factor (n=1), ovulatory disorder (n=3), endometriosis (n=1) and mixed causes (n=3).

[c]Continuous variables were compared by Wilcoxon rank sum test and categorical variables by Fisher's exact test.

[d]Data was missing for one participant in the male factor infertility group.

[e]Primary: female has never conceived before. Secondary: female has conceived before.

Table S4. **Endometrial microbiota characteristics by infertility causes**[a]

	Male factor (n=46)	Unexplained infertility (n=38)	Other infertility[b] (n=8)	p male factor vs. unexplained[c]	p male factor vs. unexplained + other[c]	p unexplained vs. male factor + other[c]
Alpha diversity						
Mean inverse Simpson diversity (SD)	2.68 (1.66)	2.73 (2.22)	3.45 (3.35)	0.819	0.891	0.803
Mean Chao1 diversity (SD)	14.22 (17.52)	15.63 (26.30)	10.50 (6.78)	0.846	0.891	0.852
Untargeted ANCOM-BC results[c, d]	---	---	---	---	---	---
Targeted bacterial groups[c, e]						
Lactobacillus genus				0.634	0.537	0.733
Median RA (IQR)	93.5 (54.1-98.5)	96.9 (18.9-99.7)	96.5 (64.4-99.8)			
95% CI						
Mean RA (SD)	71.7 (36.6)	65.4 (42.3)	74.5 (39.3)			
95% CI	60.8-82.5	51.4-79.3	41.7-100			
L. crispatus				0.910	0.688	0.871
Median RA (IQR)	0 (0-78.0)	0 (0-98.9)	0 (0-23.6)			
95% CI	0-57.2	0-65.8	0-98.6			
Mean RA (SD)	35.6 (41.7)	33.8 (46.4)	24.1 (44.7)			
95% CI	23.2-48.0	18.5-49.0	0-61.5			
L. iners				0.758	0.869	0.702
Median RA (IQR)	0 (0-40.1)	0 (0-31.6)	10.8 (0-70.7)			
95% CI	0-15.0	0-5.5	0-92.1			
Mean RA (SD)	24.3 (36.6)	22.9 (37.8)	32.9 (40.7)			
95% CI	13.5-35.2	10.5-35.4	0-66.9			
Other lactobacilli				0.951	0.674	0.795
Median RA (IQR)	0 (0-3.2)	0.1 (0-2.7)	6.3 (0-12.1)			
95% CI	0-1.4	0-1.5	0-99.8			
Mean RA (SD)	11.7 (27.8)	8.6 (21.9)	17.5 (33.8)			
95% CI	3.5-20.0	1.4-15.8	0-45.8			
Gardnerella genus				0.529	0.762	0.386
Median RA (IQR)	0 (0-0)	0 (0-26.5)	0 (0-0)			
95% CI	0-0	0-0	0-31.3			
Mean RA (SD)	8.7 (21.3)	17.1 (31.3)	3.9 (11.0)			
95% CI	2.3-15.0	6.9-27.4	0-13.1			
Other BV-anaerobes				0.377	0.595	0.258
Median RA (IQR)	0 (0-1.9)	0.1 (0-1.9)	0 (0-1.5)			

95% CI	0-0.2	0-0.9	0-20.3			
Mean RA (SD)	7.3 (17.8)	6.7 (15.2)	3.3 (7.2)			
95% CI	2.0-12.6	1.7-11.7	0-9.3			
Other bacteria (IQR)[f]				0.470	0.660	0.372
Median RA (IQR)	1.8 (0-10.2)	0.7 (0-6.4)	3.5 (0.2-24.6)			
95% CI	0.3-6.3	0.1-3.9	0-73.7			
Mean RA (SD)	12.4 (22.6)	10.8 (23.1)	18.2 (27.8)			
95% CI	5.7-19.1	3.2-18.4	0-41.5			
RA subgroups of bacterial groups						
Lactobacillus genus RA, n (%)				0.576	0.606	0.549
≤10%	7 (15.2)	9 (23.7)	1 (12.5)			
11-89%	13 (28.3)	8 (21.1)	2 (25.0)			
≥90%	26 (56.5)	21 (55.3)	5 (62.5)			
L. crispatus RA, n (%)				0.061	**0.030**	0.115
≤10%	24 (52.2)	24 (63.2)	6 (75.0)			
11-89%	11 (23.9)	2 (5.3)	0			
≥90%	11 (23.9)	12 (31.6)	2 (25.0)			
L. iners RA, n (%)				0.897	1.000	0.669
≤10%	29 (63.0)	26 (68.4)	4 (50.0)			
11-89%	11 (23.9)	7 (18.4)	3 (37.5)			
≥90%	6 (13.0)	5 (13.2)	1 (12.5)			
Other lactobacilli RA, n (%)				0.811	0.845	0.708
≤10%	38 (82.6)	32 (84.2)	5 (62.5)			
11-89%	5 (10.9)	5 (13.2)	2 (25.0)			
≥90%	3 (6.5)	1 (2.6)	1 (12.5)			
>10% *Gardnerella* genus RA, n (%)	8 (17.4)	10 (26.3)	1 (12.5)	0.424	0.607	0.302
>10% Other BV-anaerobes RA, n (%)	7 (15.2)	6 (15.8)	1 (12.5)	1.000	1.000	1.000
>10% Other bacteria RA, n (%)[c]	12 (26.1)	9 (23.7)	3 (37.5)	1.000	1.000	0.810
>10% BV-anaerobes or other bacteria RA, n (%)	18 (39.1)	13 (34.2)	3 (37.5)	0.658	0.829	0.668

BV, bacterial vaginosis; CI, confidence interval; IQR, interquartile range; NS, not significant; RA, relative abundance; SD, standard deviation.

[a]Only samples with ≥100 16S rRNA sequencing reads (V1-V2 region) were included.

[b]Other causes of infertility are tubal factor (n=1), ovulatory disorder (n=3), endometriosis (n=1) and mixed causes (n=3).

[c] ANCOM-BC analyses were corrected for multiple testing and were used to identify individual taxa with significantly different RAs in comparison groups in an untargeted manner. In the targeted analyses (using prespecified bacterial groups and subgroups), we assumed that the data were not normally distributed. All p-values were calculated by Wilcoxon rank sum tests only, but we are showing both median and mean RAs for illustrative purposes.

[d]None of the ANCOM-BC comparisons identified any taxa that were differentially relatively abundant between comparison groups.

[e]Relative abundances are presented as percentages of the number of reads of the taxon out of the total number of bacterial reads.

[f]The group of "other bacteria" contains skin bacteria, unresolved bacteria and minority taxa that could not be assigned to any of the other categories.

Figure S4: Endometrial microbiota compositions by infertility causes. The alpha diversity, beta diversity and relative abundances of bacterial taxa in endometrium of women with male factor infertility, unexplained infertility and other causes of infertility. **a.** Graphical visualisation of alpha diversity analysis comparing the infertility causes. Boxplots represent median alpha diversity by Chao1 index (upper row) and Inverse Simpson index (lower row) with interquartile range. **b.** Graphical visualisation of beta diversity analysis by principal component analysis (PCA), based on centered log-ratio (clr) transformation of the relative abundances of bacterial genera in the different groups of infertility causes. **c.** Heatmaps of the 20 most relatively abundant bacterial genera in the different groups of infertility causes. The heatmaps were generated with the phyloseq and microbiome packages in R studio (v4.1.0). For **d** and **e**, see next page.

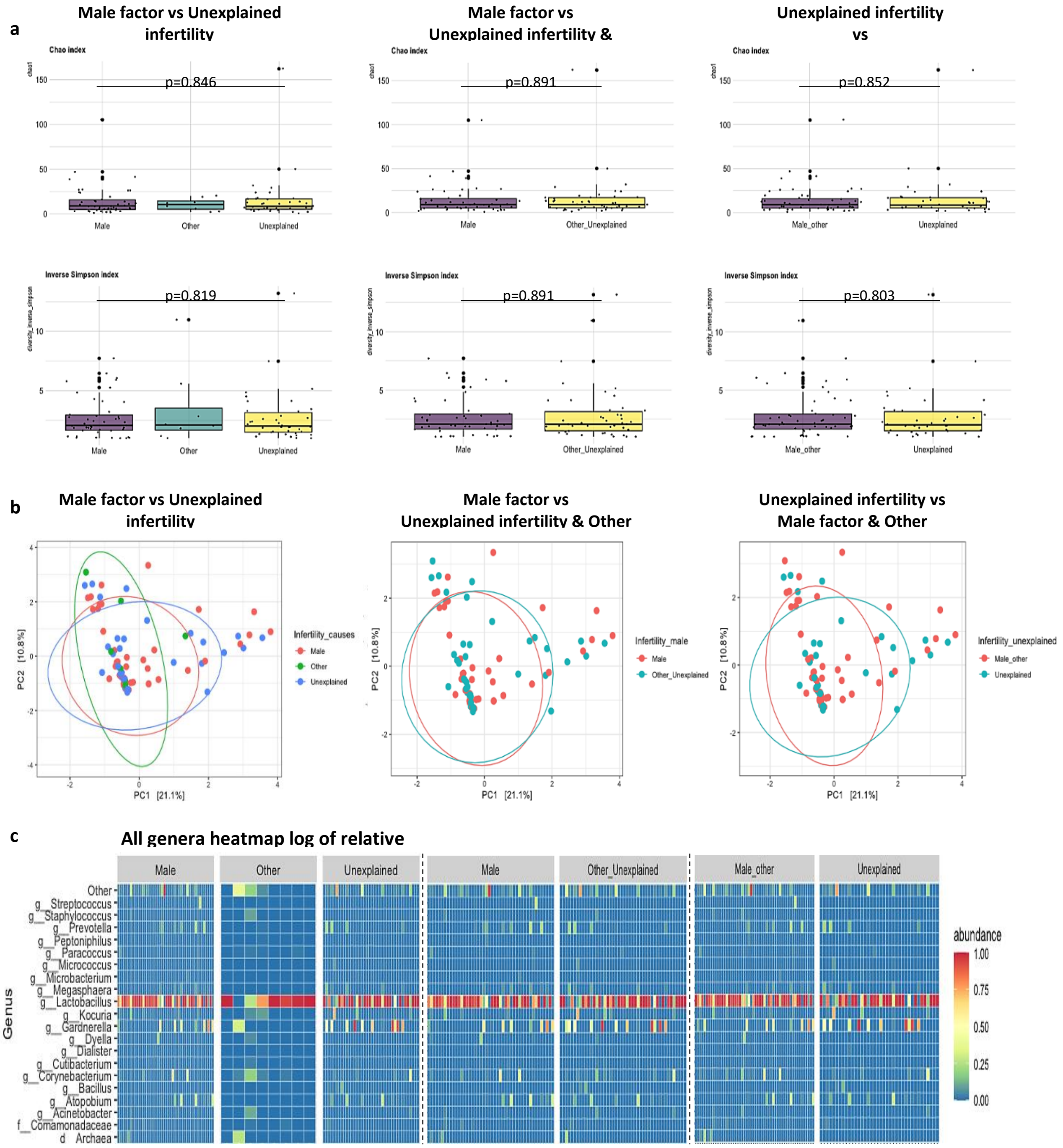
a
Male factor vs Unexplained infertility
Male factor vs Unexplained infertility &
Unexplained infertility vs
Chao index
p=0.846
p=0.891
p=0.852
Male
Other
Unexplained
Other_Unexplained
Male_other
Inverse Simpson index
p=0.819
p=0.891
p=0.803
b
Male factor vs Unexplained infertility
Male factor vs Unexplained infertility & Other
Unexplained infertility vs Male factor & Other
PC1 [21.1%]
PC2 [10.8%]
Infertility_causes
Infertility_male
Infertility_unexplained
c
All genera heatmap log of relative
Genus
abundance
Other
g__Streptococcus
g__Staphylococcus
g__Prevotella
g__Peptoniphilus
g__Paracoccus
g__Micrococcus
g__Microbacterium
g__Megasphaera
g__Lactobacillus
g__Kocuria
g__Gardnerella
g__Dyella
g__Dialister
g__Cutibacterium
g__Corynebacterium
g__Bacillus
g__Atopobium
g__Acinetobacter
f__Comamonadaceae
d__Archaea

Figure S4 (continued). Stacked bar graphs of mean (**d**) and median (**e**) relative abundances of prespecified bacterial groups in the male factor infertility, unexplained infertility and other causes of infertility groups. BV, bacterial vaginosis; *G. vaginalis, Gardnerella vaginalis*; *L. crispatus, Lactobacillus crispatus*; *L. iners, Lactobacillus iners*.

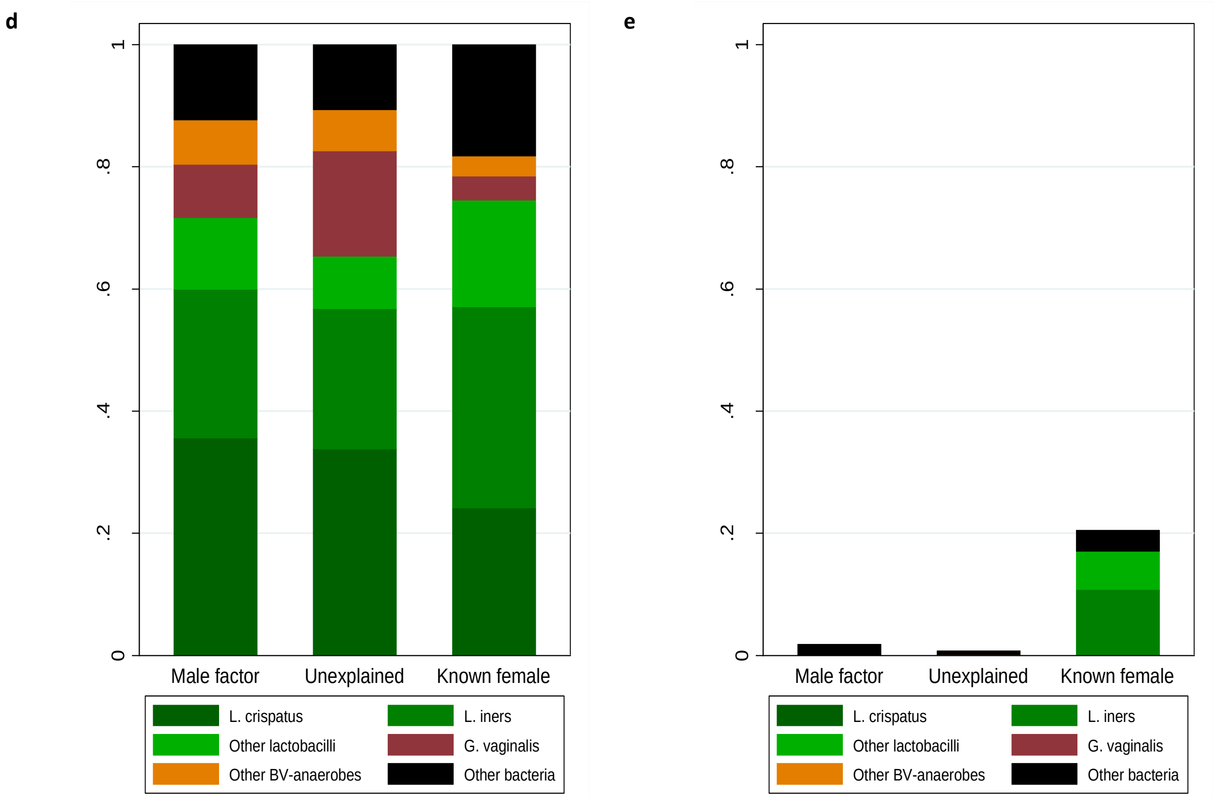

**Table S5.** Participant characteristics by infertility type[a]			
	**Primary (n=52)**[b]	**Secondary (n=40)**[b]	**p-value**[c]
Median female age in yrs (IQR)	33.4 (28.4-37.4)	36.8 (33.3-40.0)	**0.001**
Median female BMI in kg/m^2 (IQR)[d]	23.2 (21.6-25.2)	24.1 (21.8-26.1)	0.237
Median duration of infertility in months (IQR)	32.0 (24.0-48.8)	26.5 (21.3-37.5)	0.112
Female smokers, n (%)	6 (11.5)	2 (5.0)	0.458
Cause of infertility, n (%)			0.224
Male factor	30 (57.7)	16 (40.0)	
Unexplained	19 (36.5)	19 (47.5)	
Other[e]	3 (5.8)	5 (12.5)	
Median # previous embryo transfers per participant (IQR)	1.0 (1.0-3.0)	2.0 (1.0-3.0)	0.269
Pregnancy outcome during follow-up, n (%)			0.835
Live birth	22 (42.3)	16 (40.0)	
No live birth	30 (57.7)	24 (60.0)	

BMI, body mass index; IQR, interquartile range.
[a]Only samples with ≥100 16S rRNA sequencing reads (V1-V2 region) were included.
[b]Primary: female has never conceived before. Secondary: female has conceived before.
[c]Continuous variables were compared by Wilcoxon rank sum test and categorical variables by Fisher's exact test.
[d]Data was missing for one participant in the secondary infertility group.
[e]Other causes of infertility are tubal factor (secondary infertility: n=1), ovulatory disorder (primary infertility: n=1, secondary infertility: n=2), endometriosis (secondary infertility: n=1) and mixed causes (primary infertility: n=2; secondary infertility: n=1).

**Table S6.** **Endometrial microbiota characteristics by infertility type[a]**			
Alpha diversity	**Primary (n=52)[b]**	**Secondary (n=40)[b]**	**p-value[c]**
Mean inverse Simpson diversity (SD)	2.90 (2.17)	2.60 (1.95)	0.503
Mean Chao1 diversity (SD)	15.37 (23.39)	13.33 (17.47)	0.518
Untargeted ANCOM-BC results[c, d]			
Mean RA *Gardnerella* genus (SD)	6.1 (±18.1)	19.2 (±31.6)	**0.030**
Targeted bacterial groups[c, d]			
Lactobacillus genus			0.328
Median RA (IQR)	95.2 (63.4-99.3)	85.3 (12.5-99.7)	
95% CI	80.1-98.6	26.8-98.7	
Mean RA (SD)	75.3 (35.0)	61.6 (42.9)	
95% CI	65.5-85.0	47.9-75.3	
L. crispatus			**0.004**
Median RA (IQR)	35.0 (0-98.2)	0 (0-4.6)	
95% CI	0-92.4	0-0	
Mean RA (SD)	45.5 (45.7)	18.7 (35.8)	
95% CI	32.8-58.2	7.2-30.2	
L. iners			0.808
Median RA (IQR)	0 (0-39.1)	0 (0-58.2)	
95% CI	0-5.5	0-19.4	
Mean RA (SD)	23.6 (36.6)	25.8 (38.3)	
95% CI	13.4-33.8	13.5-38.0	
Other lactobacilli			0.150
Median RA (IQR)	0 (0-2.4)	0.6 (0-10.5)	
95% CI	0-0.6	0-5.2	
Mean RA (SD)	6.2 (18.2)	17.1 (32.6)	
95% CI	1.1-11.3	6.7-27.5	
Gardnerella genus			0.051
Median RA (IQR)	0 (0-0)	0 (0-34.6)	
95% CI	0-0	0-2.1	
Mean RA (SD)	6.1 (18.1)	19.2 (31.6)	
95% CI	1.0-11.1	9.1-29.3	
Other BV-anaerobes			0.260
Median RA (IQR)	0 (0-0.2)	0 (0-5.3)	
95% CI	0-0.1	0-1.8	
Mean RA (SD)	5.8 (16.3)	7.9 (15.7)	
95% CI	1.3-10.3	2.9-12.9	
Other bacteria (IQR) [e]			0.289
Median RA (IQR)	1.3 (0.1-16.3)	0.6 (0-10.0)	
95% CI	0.6-4.7	0.1-6.3	
Mean RA (SD)	12.9 (23.0)	11.4 (23.5)	
95% CI	6.5-19.3	3.9-18.9	
RA subgroups of bacterial groups			
Lactobacillus genus RA, n (%)			0.241
≤10%	7 (13.5)	10 (25.0)	
11-89%	12 (23.1)	11 (27.5)	
≥90%	33 (63.5)	19 (47.5)	
L. crispatus RA, n (%)			**0.009**
≤10%	24 (46.2)	30 (75.0)	

11-89%	8 (15.4)	5 (12.5)	
≥90%	20 (38.5)	5 (12.5)	
L. iners RA, n (%)			0.782
≤10%	35 (67.3)	24 (60.0)	
11-89%	11 (21.2)	10 (25.0)	
≥90%	6 (11.5)	6 (15.0)	
Other lactobacilli RA, n (%)			0.102
≤10%	46 (88.5)	29 (72.5)	
11-89%	5 (9.6)	7 (17.5)	
≥90%	1 (1.9)	4 (10.0)	
>10% *Gardnerella* genus RA, n (%)	6 (11.5)	13 (32.5)	**0.019**
>10% Other BV-anaerobes RA, n (%)	6 (11.5)	8 (20.0)	0.381
>10% Other bacteria RA, n (%)[e]	14 (26.9)	10 (25.0)	1.000
>10% Other BV-anaerobes or other bacteria RA, n (%)	18 (34.6)	16 (40.0)	0.665

BV, bacterial vaginosis; CI, confidence interval; IQR, interquartile range; RA, relative abundance; SD, standard deviation.

[a]Only samples with ≥100 16S rRNA sequencing reads (V1-V2 region) were included.

[b]Primary: female has never conceived before. Secondary: female has conceived before.

[c]ANCOM-BC analyses were corrected for multiple testing and were used to identify individual taxa with significantly different RAs in comparison groups in an untargeted manner. In the targeted analyses (using prespecified bacterial groups and subgroups), we assumed that the data were not normally distributed. All p-values were calculated by Wilcoxon rank sum tests only, but we are showing both median and mean RAs for illustrative purposes.

[d]Relative abundances are presented as percentages of the number of reads of the taxon out of the total number of bacterial reads in each sample.

[e]The group of "other bacteria" contains skin bacteria, unresolved bacteria and minority taxa that could not be assigned to any of the other categories.

Figure S5: Endometrial microbiota composition by infertility type. Comparison of endometrial microbiota composition and diversity between the primary and secondary infertility groups. **a.** Graphical visualisation of alpha diversity analysis. Boxplots represent median alpha diversity by Chao1 index and Inverse Simpson index with interquartile range. **b.** Graphical visualisation of beta diversity analysis by principal component analysis (PCA), based on centered log-ratio (clr) transformation of the relative abundances of genera. **c.** Heatmap representing the 20 most relatively abundant bacterial genera. Each column represents a subject, whereas each row represents a genus. The heatmaps were generated with the phyloseq and microbiome packages in R studio (v4.1.0). For **d** and **e**, see next page.

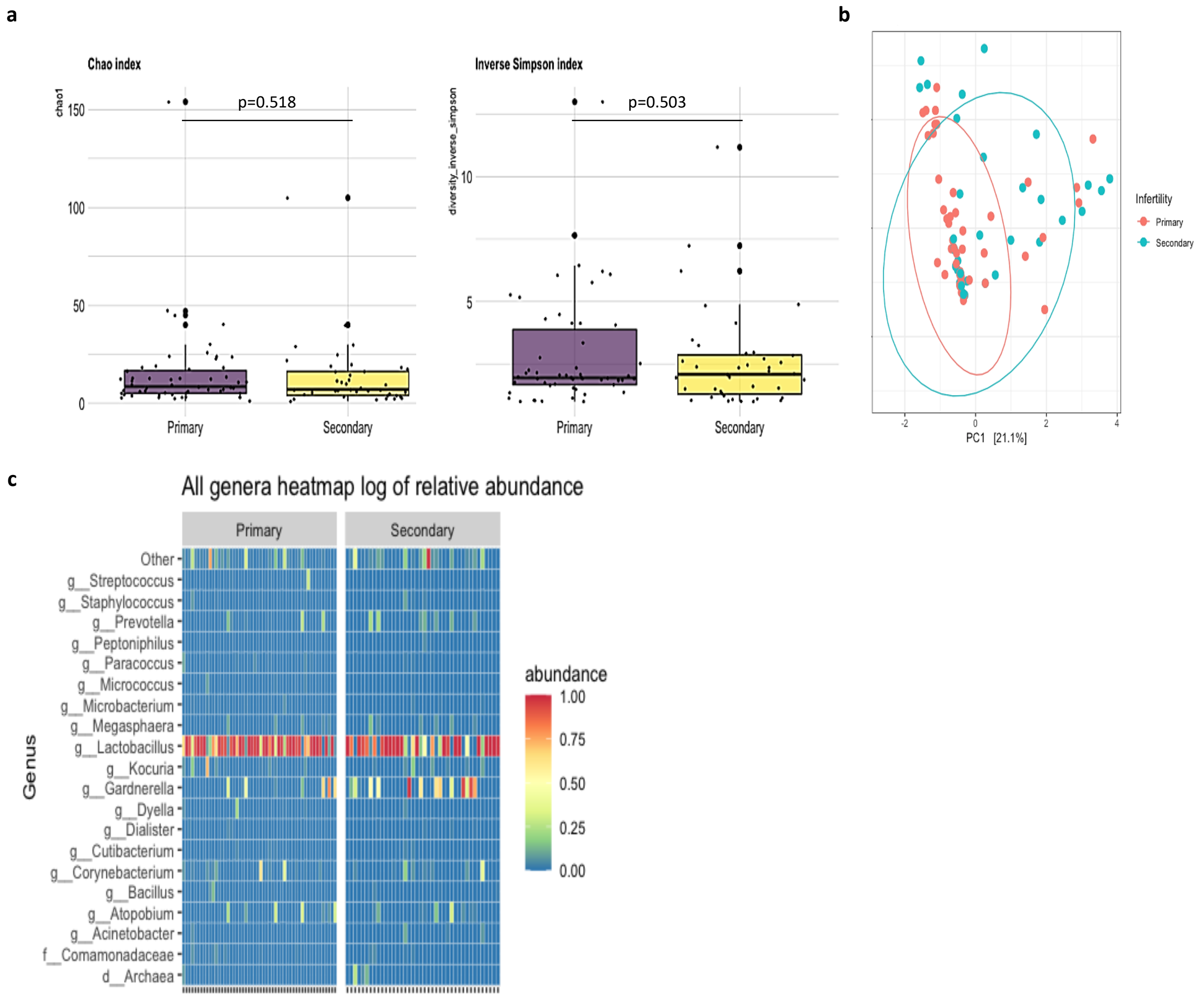

Figure S5 (continued). Stacked bar graphs of mean (**d**) and median (**e**) relative abundances of prespecified bacterial groups in the primary and secondary infertility groups. BV, bacterial vaginosis; *G. vaginalis, Gardnerella vaginalis*; *L. crispatus, Lactobacillus crispatus*; *L. iners, Lactobacillus iners.*

d

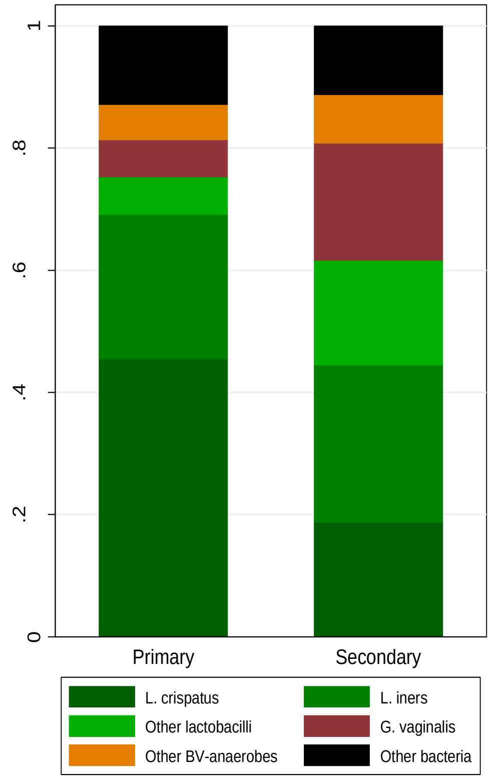

e

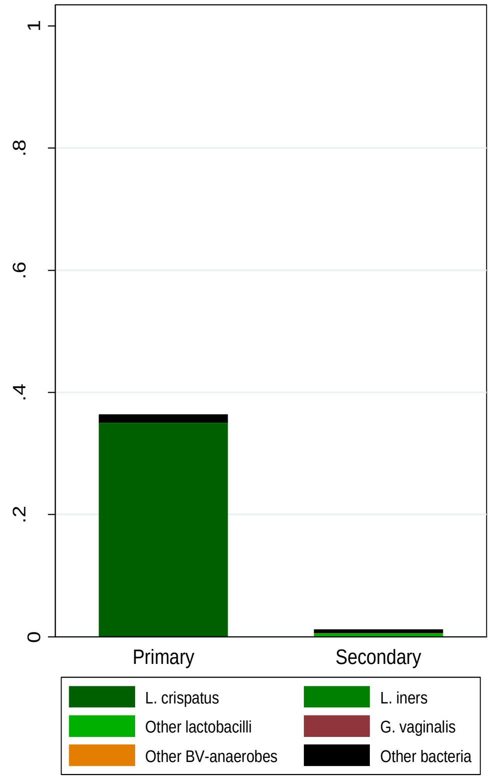

Supplement: Supplementary file 1 — Supplementary Information 1. [file 41598_2023_30591_MOESM1_ESM.pdf]
